# Supplementary figures and images for: Gp41-targeted antibodies restore infectivity of a fusion-deficient HIV-1 envelope glycoprotein
Source: PLoS Pathog. 2020 May 11;16(5):e1008577. doi: 10.1371/journal.ppat.1008577 (PMC7241850; doi:10.1371/journal.ppat.1008577)

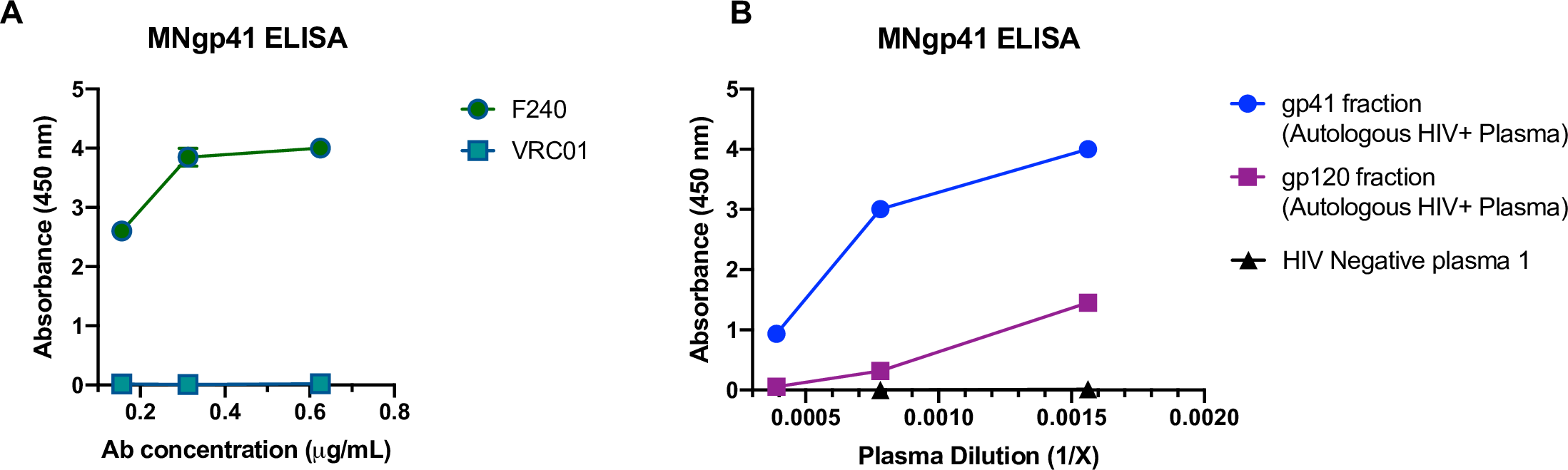

Supplement: S1 Fig — Binding of (A) gp41-specific antibody F240 and gp120-specific antibody VRC01 to plates coated with MNgp41 protein. MNgp41 binding of (B) autologous HIV-1-positive plasma (4.6 ypi) depleted of gp120 antibodies by RSC3core protein: “gp41 fraction”, or gp120 antibodies eluted from the RSC3core protein “gp120 fraction” and HIV-1-negative plasma (control). Samples were tested in duplicate. Data represents mean values and error bars indicate SEM. (TIF) [file ppat.1008577.s001.tif]

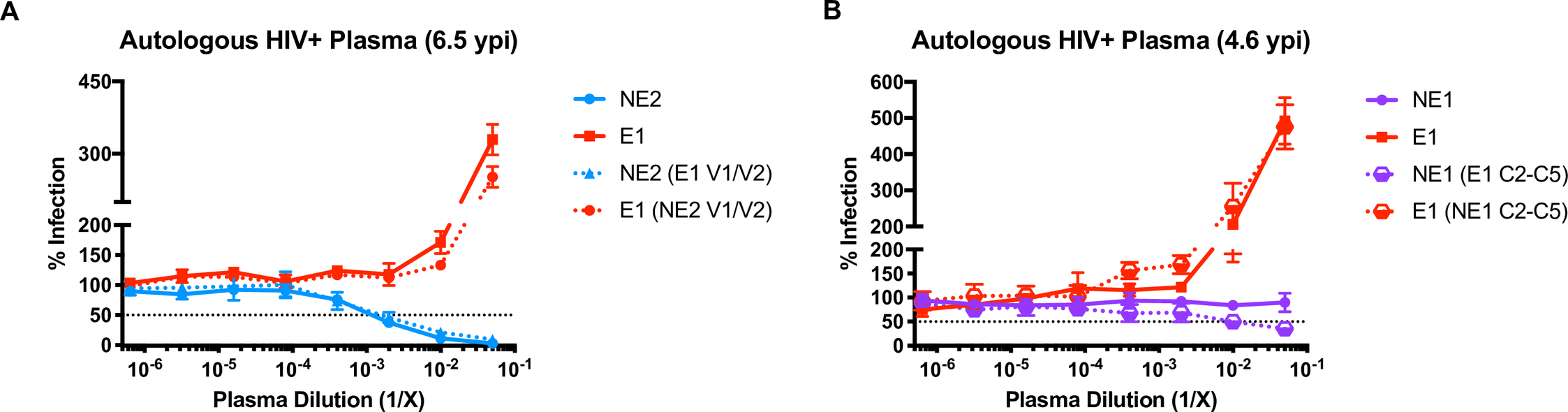

Supplement: S2 Fig — Pseudoviruses expressing (A) V1/V2 chimeras of Envs E1 and NE2 were tested for infection of TZM-bl cells in the presence of HIV-1-positive plasma (6.5 ypi). (B) Env chimeras of E1 and NE1 swapping the C2, C3, V3, C4, V4 and C5 regions were tested for infection of TZM-bl cells in the presence of HIV-1-positive plasma (4.6 ypi). (TIF) [file ppat.1008577.s002.tif]

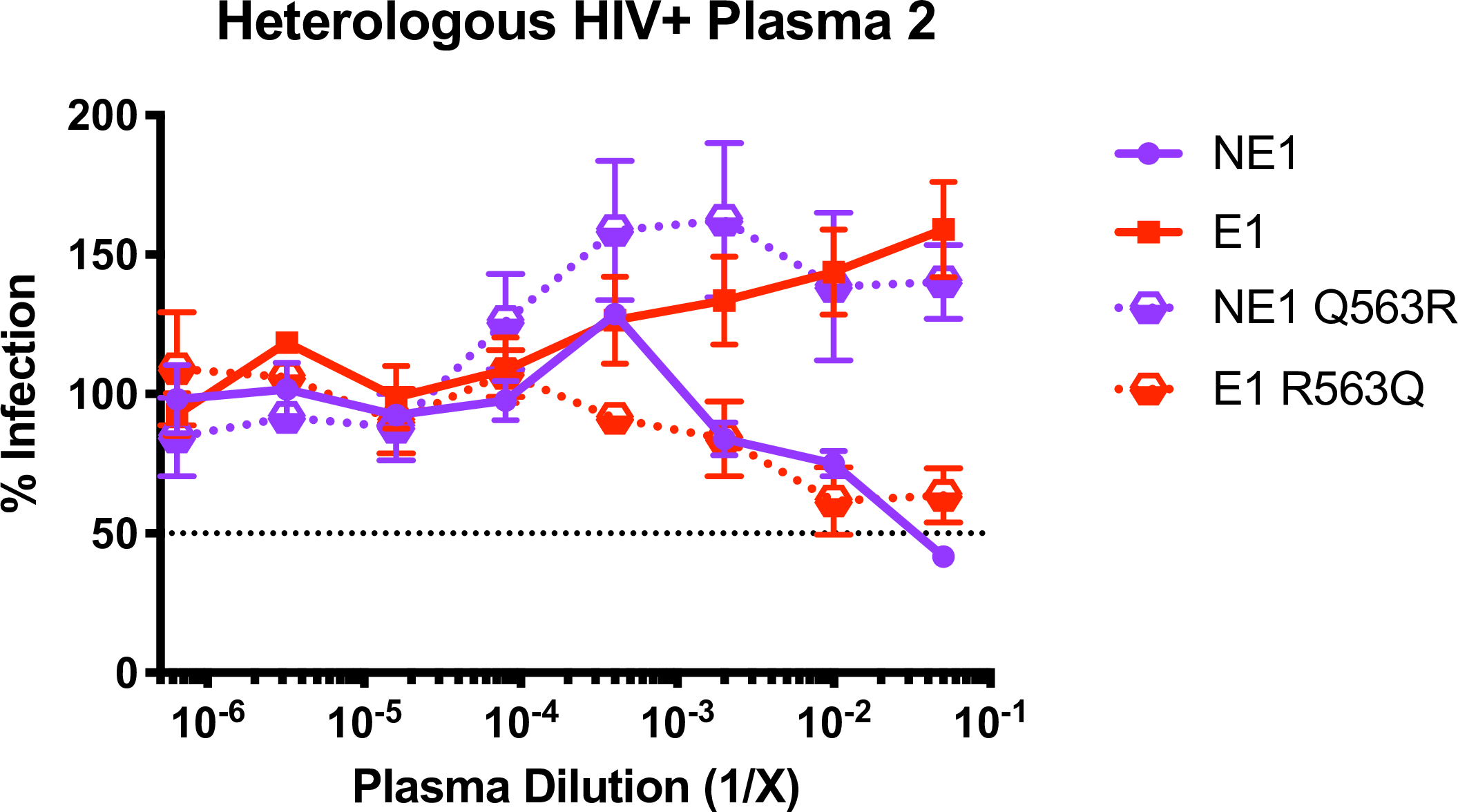

Supplement: S3 Fig — Comparative infection of TZM-bl cells in the presence of a second heterologous HIV-1 positive plasma sample (Heterologous HIV+ Plasma 2) by NE1, NE1 Q563R, E1 and E1 R563Q viruses. All assays were done in triplicate. Data are represented as mean values; error bars indicate SEM. The dotted line indicates 50% infection. (TIF) [file ppat.1008577.s003.tif]

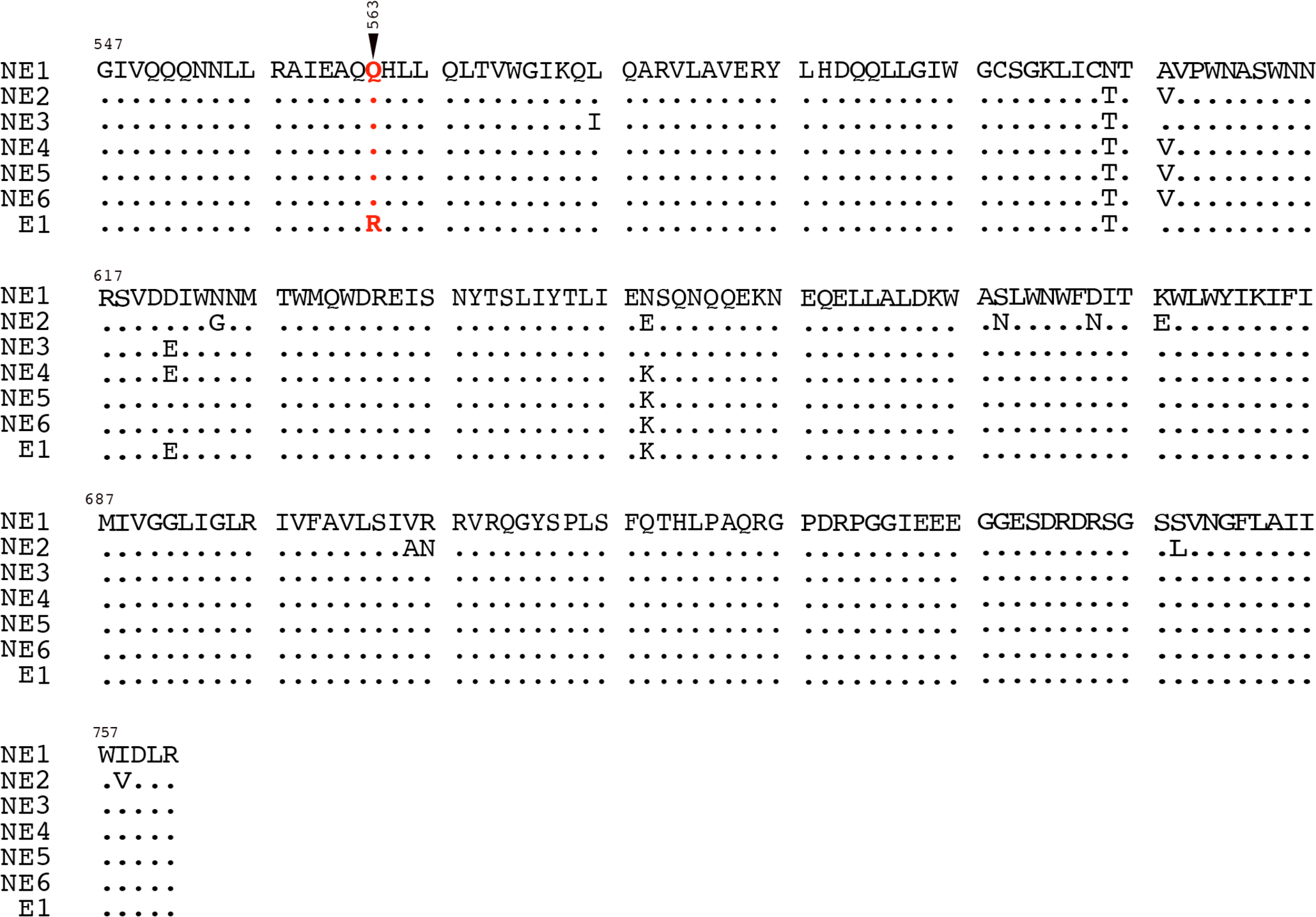

Supplement: S4 Fig — Amino acid alignment of NE1-NE6 and E1 is shown. HXB2 numbering is shown on the top left corner of each section. Dots indicate sequence identity. Non-conserved residues are displayed. The Q563R change unique to E1 is shown in red. (TIF) [file ppat.1008577.s004.tif]

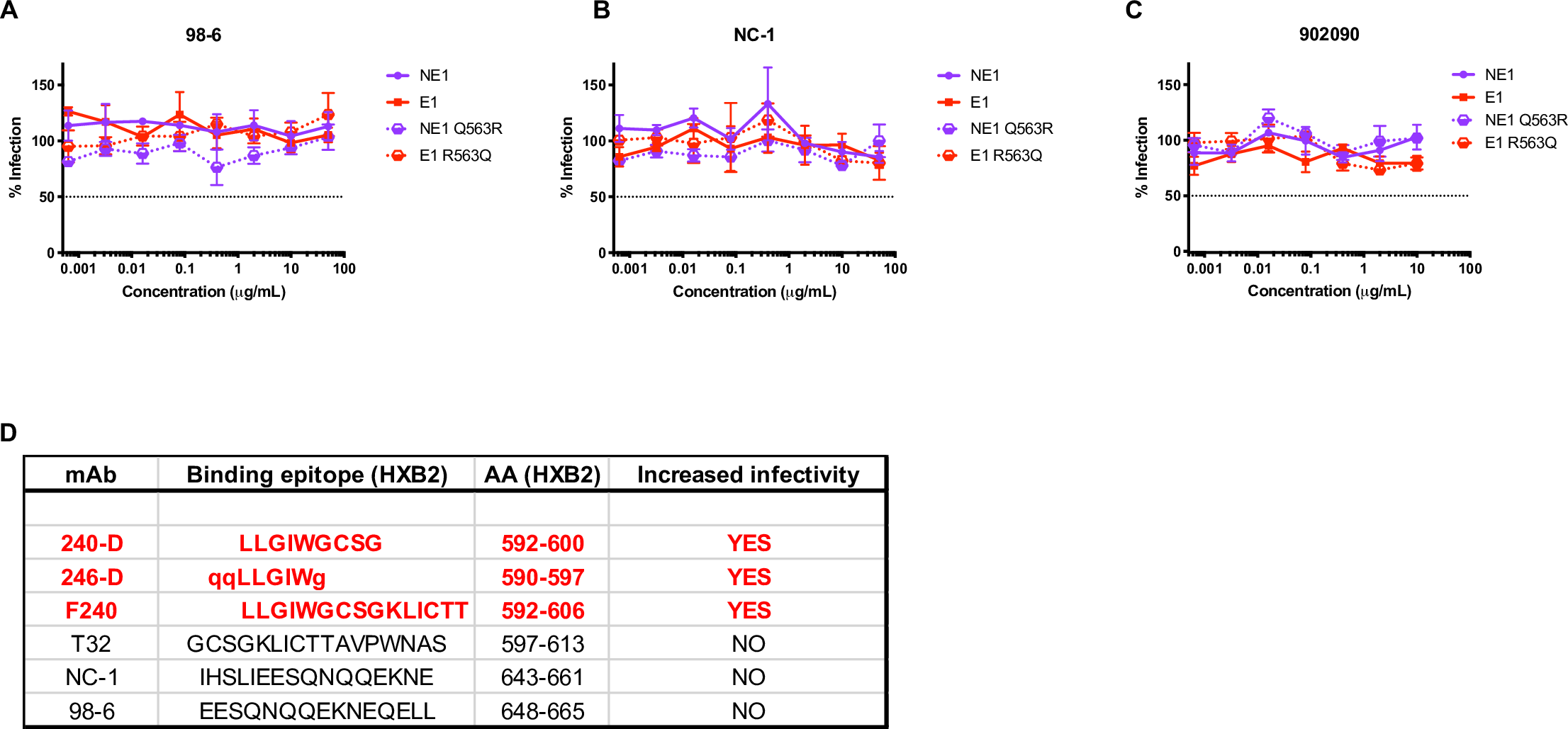

Supplement: S5 Fig — Infection of TZM-bl cells by NE1, NE1 Q563R, E1 and E1 R563Q viruses was tested in the presence of anti-cluster II mAbs (A) 98–6, (B) NC-1 and V2-targeting antibody (C) 902090. (D) The gp41-binding epitopes of the HR1- and HR2-targeting antibodies tested for ability to increase infectivity. (TIF) [file ppat.1008577.s005.tif]

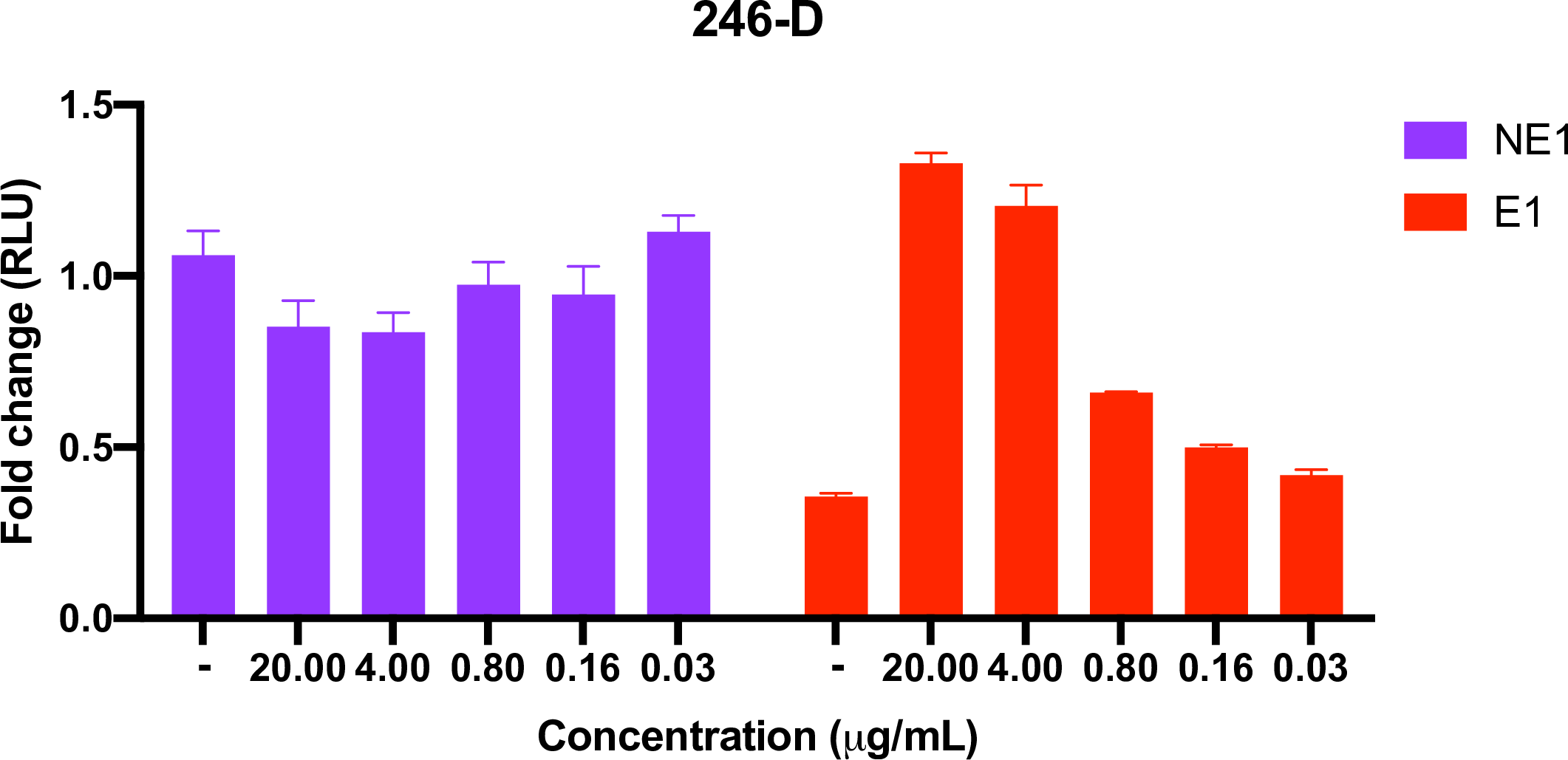

Supplement: S6 Fig — Fold change in infection of TZM-bl cells by E1 and E1 R563Q viruses with different amounts of 246-D is shown. All assays were done in triplicate. Data are represented as mean values; error bars indicate SEM. (TIF) [file ppat.1008577.s006.tif]

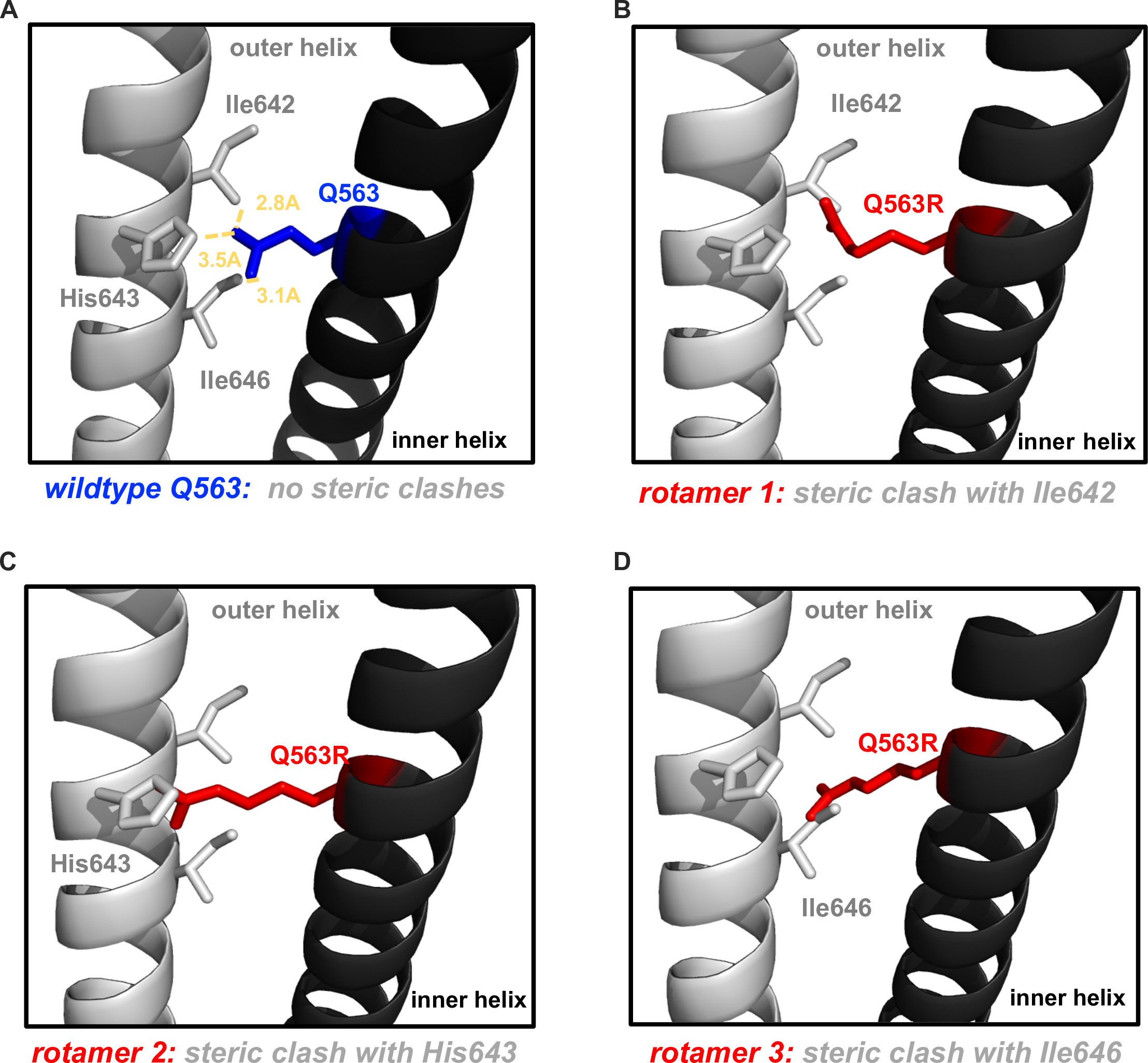

Supplement: S7 Fig — Potential interactions of (A) Q563 within HR1 (inner helix) with residues in HR2 (outer helix) are shown. Dotted lines indicate atomic distances between these residues. Potential steric clashes of Q563R with (B) isoleucine at position 642, (C) histidine at position 643 and (D) isoleucine at position 646 within HR2 are depicted. All images were created using the PyMOL Molecular Graphics System, Version 2.0 Schrödinger, LLC, using PDB 1AIK as template [14]. (TIF) [file ppat.1008577.s007.tif]
