# Supplementary material for: Gp41-targeted antibodies restore infectivity of a fusion-deficient HIV-1 envelope glycoprotein
Source: PLoS Pathog. 2020 May 11;16(5):e1008577. doi: 10.1371/journal.ppat.1008577 (PMC7241850; doi:10.1371/journal.ppat.1008577)
Supplement: Attachment — Submitted filename: Joshi_PLOSPath_Response to Reviewers.pdf [file ppat.1008577.s009.pdf]

Point-by-Point Response to Reviewer's Comments

We thank the reviewers for their encouraging feedback on this manuscript and appreciated the insightful comments and suggestions. As requested, below we provide a point-by-point response to each of the reviewers' comments, including, where appropriate, the page and line numbers in the revised manuscript where revisions have been made. Manuscript revisions are made using 'track changes' for clarity.

Part I: Summary

Reviewer #1: ***I have a few minor comments*****:**

1. ***I would be interested to know if infection enhancement is seen with a larger panel of plasmas - two were used in this study, but then in the discussion it is stated that plasma from multiple individuals had this effect. I suppose 2 is multiple by definition, but could the authors be more specific in the text?***

   We appreciate the detailed and thoughtful comments on this manuscript. In response to this question we have now included an additional plasma sample from a second heterologous HIV-1 infected individual (Heterologous HIV+ Plasma 2). As before, this HIV+ plasma also demonstrates the ability to enhance infectivity with E1 (as well as against NE1 Q563R) but not NE1. Thus, we now observe this increased infectivity with plasma samples across different time points from three unrelated HIV-1 infected individuals (1 autologous and 2 heterologous). In addition, we have thus far not identified any HIV+ plasma that does not mediate increased infectivity of Q563R-containing Envs.

   As such, we have now included and additional figure (**S3 Fig.**) illustrating these data and have added the following statement on Page 10, Lines 208-210: "*We further tested the infectivity of these viruses in the presence of heterologous plasma from another unrelated HIV-1 infected individual (Heterologous HIV+ Plasma 2)* ***(S3 Fig.)****.*"

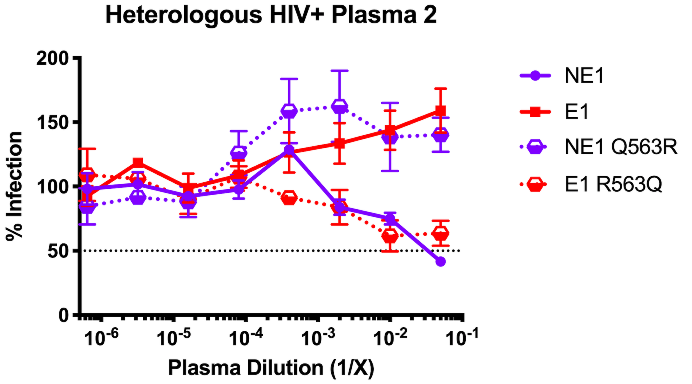

   While we would be keen to test additional plasma samples for this purpose, we are currently experiencing an 8-week lab shutdown across Harvard and MGH (as are many other places) which required ceasing all activities on this project. This shutdown would result in a minimum 3-month delay in incorporating the suggested data for our revised submission and we therefore respectfully request forgoing any additional work on this minor comment.

2. ***When starting to sort out what antibodies might be responsible for the enhancement effect, the authors fractionated plasma by using magnetic beads coated with a form of gp120. The flow-through faction contained the enhancing activity and so the conclusion is that gp41 antibodies are responsible. While this eventually proved to be true, it is unlikely that any artificial Env construct would can all gp120 antibodies – it is probably more accurate to say that their results suggest that gp41 antibodies are the ones responsible (page 8).***

   We have modified our text on Page 8, Lines 146-148 to reflect this observation as follows: "*These observations suggest that gp41-targeted antibodies mediate the increase in infection by viruses with Env E1.*"

Part II – Major Issues: Key Experiments Required for Acceptance

Reviewer #1: (No Response)

Reviewer #2:

1. ***Very few issues were observed and should be barely considered as major. The authors report that heterologous plasma could rescue the infectivity of the E1 R562Q isolates. Was the heterologous plasma characterized for the presence of Nab as suggested for the autologous?***

The heterologous plasma sample used in our original submission (Heterologous HIV+ Plasma) was not characterized for the presence of NAbs. Additionally, in our neutralization assays, this plasma did not neutralize the Env NE1 virus. This lack of neutralization of these primary Envs allowed us to differentiate the Q563R-mediated phenotype of increased infectivity from resistance to neutralization. In this revised submission, we have included neutralization data with plasma from a second unrelated HIV-1 infected individual (indicated as Heterologous HIV+ Plasma 2). This plasma is seen to neutralize Env NE1 virus, indicating the presence of NAbs. The Q563R containing Envs (E1 and NE1 Q563R) are not neutralized by this Env and show a similar 3-fold increase in infectivity at the highest plasma concentrations tested. This data is shown in a new **S3 Fig**.

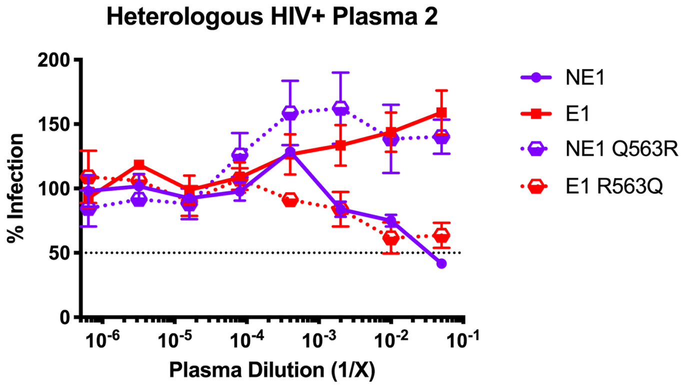

As noted above, we have now included an additional figure (**S3 Fig.**) illustrating these data and have added the following statement on Page 10, Lines 208-216 describing this effect: "*We further tested the infectivity of these viruses in the presence of heterologous plasma from another unrelated HIV-1 infected individual (Heterologous HIV+ Plasma 2)* ***(S3 Fig.)****. Unlike Heterologous HIV+ Plasma 1* ***(Fig. 3A)****, Heterologous HIV+ Plasma 2 exhibited neutralization of the Env NE1 virus. Both Env E1 and Env NE1 Q563R viruses were not neutralized by this plasma, but instead exhibited increased infectivity. On the same lines, the R→Q substitution in Env E1 R563Q virus did not support increased infectivity, and reverted to the phenotype seen with Env NE1 virus, indicating that the Q563R change mediated increased infectivity even in the presence of neutralizing antibodies in HIV positive plasma* ***(S3 Fig)****.*"

2. ***Related to the above comment, we observed a statement in the discussion stating that " heterologous plasma from multiple donors" (line 424) was used. However, this is not reflected in any of the figures.***

As noted above we have now added in neutralization data with plasma from a second HIV-1 infected individual (indicated as Heterologous HIV+ Plasma 2). This data is shown in the new **S3 Fig**.

3. ***The independence from Fc Receptor should be supported by the utilization of Fab to enhance the infection.***

We observed the increased infectivity of Q563R-containing Envs in TZM-bl cells. Multiple reports show that TZM-bl cells, on their own, are devoid of Fc-Receptors [1-3]. We further used a commercially available Fc-Receptor blocker, as an additional control in these TZM-bl cells. We believe these data, taken together, strongly suggest that Fc-Receptors do not play a role in the increased infectivity observed in our experiments.

We agree that using Fab fragments would further support the observation of Fc-Receptor independence of the observed increased infectivity. However, while we have been trying to express these fragments, the expression of the monoclonal antibodies has been extremely low precluding our ability to address this question at the moment. As a result of our laboratory shut down, development of Fab fragments to address this question would result in a substantial delay in the preparation of the final manuscript and we therefore respectfully request forgoing any additional work on this comment.

Reviewer #3:

1. ***The description of a mutation that relies on the host antibody responses to achieve efficient infection is quite interesting. The authors note that related phenomena have been described in the context of drug treatment (T-20). From the standpoint of the multitude of ways in which HIV can overcome host immune responses this observation stands out. It is also a bit discouraging in that it makes one wonder if there are almost unlimited ways in which the virus can evolve escape mechanisms. On line 381 the authors indicate that "for the first time" they have identified a mechanism whereby a viral defect is repaired by the host immune response. I would suggest that this statement be circumscribed to indicate more specifically that this a first in terms of a host humoral immune response that improves the function of the envelope.***

   We appreciate the reviewer's insight into this surprising novel mechanism by which HIV-1 can evolve to survive and have slightly modified our text as requested to indicate the role of the host's humoral immune response. Importantly, previous literature implicates some HIV-specific antibodies in enhancing HIV-1 infection in vitro; thus, this would not be the first report of the humoral immune response improving viral function [4, 5]. It is important to note that these previous studies by Williams et al., and Robinson et al., show the enhancement of infection of all viruses tested, and do so in a complement-dependent manner, while the increased infectivity observed with gp41 antibodies in our data is specific to Envs with a Q563R change, and is independent of complement.

   As suggested we have thus changed our text, Page 18, Lines 389-392, to: "*Our study demonstrates, we believe for the first time, a mechanism whereby a viral defect is alleviated by the host's humoral immune response, specifically the Q563R-determined impaired infectivity of E1 viruses being restored in the presence of anti-HR1 antibodies*".

2. ***All of the infection assays are carried out with TZM-bl cells, including those used to measure the sensitivity of the envs to gp41 mAbs. I am concerned that these observations might not reflect the impact of arginine 563 in a virus that is targeting primary CD4+ T cells. Given the substantial effort that went into identifying and characterizing this interesting mutation, I wonder why the authors don't want to find out if their observations are relevant to an infection system that is a little more reflective or reality.***

   We appreciate this suggestion to test our observations in a more realistic infection system. We agree that this information would be a great addition to the manuscript. However, as noted above,

the current COVID-19 pandemic has forced a lab shutdown across Harvard and MGH for the next 8 weeks. Assuming we will be allowed to return to work after 8 weeks, we would require 2-3 weeks to get the lab up and running, especially cell cultures/cell lines, followed by 4 weeks to complete this assay.

The proposed experiment is definitely feasible, but would result in a 4-month delay at best in submitting the revised manuscript. Therefore, we would respectfully request forgoing any additional work to address this comment.

Part III – Minor Issues: Editorial and Data Presentation Modifications

Reviewer #1: (No Response)

Reviewer #2: (No Response)

Reviewer #3: (No Response)

References:

1. Cheeseman, H.M., et al., *Broadly Neutralizing Antibodies Display Potential for Prevention of HIV-1 Infection of Mucosal Tissue Superior to That of Nonneutralizing Antibodies.* J Virol, 2017. **91**(1).
2. Richardson, S.I., et al., *IgG3 enhances neutralization potency and Fc effector function of an HIV V2-specific broadly neutralizing antibody.* PLOS Pathogens, 2019. **15**(12): p. e1008064.
3. Perez, L.G., et al., *Utilization of immunoglobulin G Fc receptors by human immunodeficiency virus type 1: a specific role for antibodies against the membrane-proximal external region of gp41.* Journal of virology, 2009. **83**(15): p. 7397-7410.
4. Williams, K.L., et al., *Identification of HIV gp41-specific antibodies that mediate killing of infected cells.* PLOS Pathogens, 2019. **15**(2): p. e1007572.
5. Robinson, W.E., Jr., et al., *Two immunodominant domains of gp41 bind antibodies which enhance human immunodeficiency virus type 1 infection in vitro.* J Virol, 1991. **65**(8): p. 4169-76.
